# Comparison of the effectiveness of oral lactase enzyme and lactose-free formula in the management of secondary lactose intolerance in persistent and severe persistent diarrhea

**DOI:** 10.12669/pjms.42.8.12258

**Published:** 2026-08

**Authors:** Fatima Ghayas Siddiqi, Heena Rais, Tayyaba Anwar, Aizaz Ali Khan

**Affiliations:** 1 Fatima Ghayas Siddiqi, FCPS Department of Pediatrics, Hamdard University Hospital, Karachi, Pakistan; 2 Heena Rais, FCPS, Department of Pediatrics, Ziauddin University, Karachi, Pakistan; 3 Tayyaba Anwar, FCPS, Department of Pediatrics, Ziauddin University, Karachi, Pakistan; 4 Aizaz Ali Khan, Department of Pediatrics, Ziauddin University, Karachi, Pakistan

**Keywords:** Diarrhea, hospital stay, lactase, lactose intolerance, stool

## Abstract

**Objective:**

To compare the effectiveness of oral lactase enzyme versus lactose-free formula in the management of secondary lactose intolerance in persistent and severe persistent diarrhea.

**Methods:**

This randomized controlled trial was conducted at the pediatric unit of Ziauddin University Hospital, Kemari, Karachi, Pakistan, from June, 2023 to July,2024. A total of 50 children were included in this study following the inclusion criteria of both genders aged 6-24 months and presented with persistent or severe persistent diarrhea due to secondary lactose intolerance. In Group-A, five drops of lactase enzyme (200 units lactase/drop)/ounce of milk were added 5-30 minutes before feeding, while in Group-B, lactose-free formula milk/diet was advised. They were monitored from day one through day five and assessed for outcome measures, comprising clinical (on day five) and laboratory assessments (stool pH and reducing substances on day six), success or failure, and duration of hospital stay (less or more than five days).

**Results:**

In a total of 50 children, the median age was 11 (9-15.3) months. There were 30 (60.0%) children who presented with vomiting, while 14 (28.0%) had severe dehydration. At day five, lactase enzyme children showed significantly less episodes of diarrhea versus lactose free formula (20.0% vs. 48.0%, p=0.037), while all other complaints were statistically similar (p>0.05). The duration of hospital stay was significantly less among children in lactase enzyme group versus lactose free formula group (p=0.001).

**Conclusion:**

The effectiveness of lactase enzyme supplementation was better than lactose-free formula in children with persistent diarrhea due to secondary lactose intolerance.

Clinical Trial Registration: NCT06827405 (https://clinicaltrials.gov).

## INTRODUCTION

Diarrhea is defined as the passage of three or more loose or liquid stools per day (or more frequent passage than is normal for the individual), according to WHO.^1^ Diarrheal illnesses in children contribute significantly to the global disease burden, with the greatest impact on children living in low and middle income (LMIC) countries with limited resources.^2^ Recent statistics show that children under the age of five experience an estimated 1.3 billion episodes of diarrhea annually, which accounts for 0.5 million or 10% of diarrheal fatalities worldwide.^3^ A large majority of acute cases due to infection mainly settle within a week, however, in LMICs, 5-10% of episodes of acute diarrhea linger on, thus becoming persistent (lasting more than 14 days) and not only posing a significant disease burden.^4^,^5^ In Pakistan, diarrhea accounts for a staggering 20-30% of the total childhood mortality in the under five years age group, making it one of the four countries in the world with the heaviest burden of deaths due to diarrhea in this age group.^6^,^7^ The huge burden of diarrheal diseases shouldered by Pakistan places it 23rd on the rung of the childhood mortality ladder by WHO.^8^ Persistent diarrhea is also the 3rd leading cause of death in children under-fives.^9^,^10^

Previous and current management trends focus heavily on prebiotics, probiotics, and dietary modifications like plant-based alternative feeding or reduction/abstinence of lactose by either the caregiver or practitioner for a prolonged period.^11^ Even though it is well understood that oral lactase enzyme can play a pivotal role in hastening recovery due to secondary lactose intolerance in persistent diarrhea, scarce local data is available in this regard.^12^ If exogenous oral lactase enzyme is found to be more effective, it would help children receive uninterrupted milk and a milk-based diet during the diarrheal episode, as the former would aid digestion and absorption of lactose, thus providing the necessary nutrients, especially calcium, and vitamins so vital in this period of rapid growth. From a larger perspective, the financial and emotional burden incurred by the family would be mitigated and the development of a malnourished child and future intellectually compromised adult would be prevented, thus contributing to the economic growth of the country in terms of able manpower, especially in LMICs. The current study was planned with the objective of comparing the effectiveness of oral lactase enzyme and lactose-free formula in the management of secondary lactose intolerance in persistent and severe persistent diarrhea.

## METHODOLOGY

This randomized clinical trial was carried out at the pediatric unit of Ziauddin University Hospital, Kemari, Karachi, Pakistan, from June, 2023 to July, 2024. Sample selection was done using a non-probability consecutive sampling technique

### Ethical Approval:

It was obtained from the ethical review committee of the institution (letter number: 0880319FSPED, dated: April 17, 2019). Informed and written consents were obtained from parents/guardians of all enrolled children.

.A sample size of 34 (17 in each group) was calculated using the OpenEpi sample size calculator, considering the expected response to lactase enzyme as 76% and to placebo as 23%^13^, keeping the confidence level at 95% and the power of the study at 80%. The sample size was increased up to 50 (25 in each group) as the number of patients presenting with diarrhea are common, being a tertiary care hospital.

### Inclusion criteria:

Children of any genders, aged between six and 24 months, presenting with persistent or severe persistent diarrhea due to secondary lactose intolerance. Patients on formula or mixed feeding, or formula/cow/buffalo milk, were considered.

### Exclusion criteria:

Severe acute malnutrition, hemodynamic instability (shock), or those who were on oral antibiotics or consumed excessive juices. Children with celiac disease, protein losing enteropathy, or irritable bowel syndrome were also excluded.

The persistent diarrhea was defined as the diarrhea lasting for more than 14 days without blood, and severe persistent diarrhea was labeled when the persistent diarrhea was accompanied by dehydration. The presence of at least two clinical features from the following: abdominal pain, vomiting, bloating, flatulence, perianal rash, and/or bloating after the ingestion of lactose or lactose-containing food substances indicated secondary lactose intolerance, confirmed on the basis of laboratory assessments of stool pH ≤ 6, reducing substances positive, and occult blood negative.

Children fulfilling eligibility criteria were subjected to demographic, clinical and physical evaluation. To collect stool samples, caretakers were provided containers and taught how to collect the stool, uncontaminated with water or urine, without coming in contact with the diaper or toilet paper. Contaminated stool samples were discarded, and stool collection was tried again in clean containers. Fresh stool samples (minimum five grams of the solid as well as the liquid portion equivalent to a 5ml spoon) were collected under the careful supervision of the health care team. All the samples were put in sterile, airtight, and properly labeled containers and, by maintaining low temperatures (< 40°F) throughout, were sent to the institutional laboratory within half an hour of being passed. The stool samples were tested for acidity, and if the pH was found to be six or less, then a stool for reducing substances (a screening test used commonly in resource-poor countries) was performed using the Benedict’s solution (semi-quantitative analysis). First, the stool was removed from the container, and an equal volume of distilled water was added to it. The resultant mixture was then homogenized and centrifuged for 10 minutes, after which 2.5 ml of Benedict’s solution (made from sodium citrate and copper sulphate) was added to 10 drops of this supernatant in a test tube and was then heated in a boiling bath of water for another 10 minutes. A color change from blue (no sugar) to orange indicated excessive carbohydrates in the stool. The stool was also tested for pus cells, infective microorganisms (via culture), and ova/parasites.

A total of 50 children who had an acidic stool, positive for reducing substances, were enrolled for this study. Children were then randomly assigned to two groups using the lottery method. In Group-A, five drops of exogenous oral lactase enzyme (containing 200 units lactase/drop) in per ounce of milk were added 5-30 minutes before feeding to the regular type and quantity of milk normally taken by the patient or before any dairy foodstuff and the former was then stored in the refrigerator. In Group-B, milk and dairy products were totally avoided, and only lactose-free formula milk was advised along with a regular nondairy diet. Both the groups were given the recommended standard zinc supplementation, and patients with stool examination suggestive of infection (irrespective of stool culture) were treated with antibiotics as per WHO protocol. Those patients falling in moderate acute malnutrition according to their z-score were given food according to personalized diet charts. The patients were also strictly observed for signs of any allergic reactions like anaphylaxis, hives, tingling sensation of mouth, face, skin rash, or itching. All children were vigilantly supervised by the on duty pediatrition as well as the nursing staff so that they adhered to their respective treatment protocols. Children underwent monitoring thrice a day regarding their clinical manifestations from day one of intervention through day five to identify the outcomes including resolution of symptoms (i.e., frequency of stools ≤ 4, consistency of stools (Bristol Stool Chart) ≤ 5, reduction in abdominal pain (faces pain score revised -FPS-R), a score of ≤ 4, lessening in bloating (abdominal girth at the umbilicus by a non-stretchable measuring tape) ≤1, resolution of nausea/vomiting, no perianal rash or only erythema, and duration of hospital stay ≤ 5 days. On day six, a repeat stool analysis was carried out for all patients (admitted as well as discharged) for the confirmation if pH > 6 and stool for reducing substances negative. The secondary outcome was treatment failure assessed on the basis of all three conditions fulfilled, i.e., diarrhea (frequency > 4 stools, consistency > 5) lasting more than five days, stool pH ≤ 6, and hospital stays > 5 days. Patients were discharged as soon as there was clinical resolution, and the parents/caretakers were advised to continue their respective treatments and keep a notebook to document the diet as well as the lactase enzyme administration and any untoward symptom or reaction. Special detail was provided to store the lactase enzyme in an ice thermos so it remained cool and not frozen. Those in Group-A, who responded well to the lactase enzyme, continued with the former for a further eight weeks, while those who showed a response to lactose-free formula continued it for the same duration of time.

### Statistica Analysis:

Data was analyzed using “IBM SPSS Statistics” version 26.0. The quantitative data was reported as mean and standard deviation (SD), or median and interquartile range (depending upon the normality distribution). The qualitative data was expressed as frequency and percentage. Stool consistency, nausea/vomiting, abdominal pain, abdominal distension, perianal rash, stool pH, and reducing substances were compared between the two groups by applying the chi-square test or fisher’s exact test. The number of stool counts and duration of hospital stay were compared between the two groups by the independent sample t-test or Mann-Whiteny U test. For all inferential statistics, p< 0.05 was taken as significant.

## RESULTS

In a total of 50 children, the median age, weight, and length were 10 (9-15.3) months, 10 (7-11) kg, and 73 (70-77.5) cm, respectively. An abdominal pain score ≥ 4 was recorded in 28 (56.0%) children. There were 30 (60.0%) children who presented with vomiting. There were 14 (28.0%) children who had severe dehydration. Pus cells in stool were present in 4 (16.0%) patients in Group A as compared to 5 (20.0%) in Group-B. Stool culture and blood in stool were negative for all the patients enrolled. Giardia was seen in 6 (12.0%) patients. Both groups showed no statistically significant differences in terms gender (p=0.254), age (p=0.380), weight (p=0.217), and length (p=0.436). Table-I is showing comparison of baseline demographical, antropometric, and clinical characteristics of children in both study groups.

**Table-I:** Comparison of demographic, anthropometric, and clinical characteristics of children with diarrhea in study groups (N=50)

Characteristics		Groups		P-value
		Lactase enzyme (n=25)	Lactose free formula (n=25)	
Gender	Male	16 (64.0%)	13 (52.0%)	0.254
	Female	9 (36.0%)	12 (48.0%)
Age in months, median (IQR)		11 (9.00-18.50)	10 (9.00-14.50)	0.380
Weight in kg, median (IQR)		10 (7.00-11.10)	9 (7.00-10.35)	0.217
Length in cm, mean (SD)		74.56±5.66	73.32±5.49	0.436
Z-score	Median	9 (36.0%)	6 (24.0%)	0.568
	< -1SD	9 (36.0%)	9 (36.0%)	
	< -2SD	7 (28.0%)	10 (40.0%)	
Mode of feeding	Breast feeding	-	-	0.774
	Formula milk	11 (44.0%)	10 (40.0%)	
	Cow/buffalo	14 (56.0%)	15 (60.0%)	
Abdominal pain (FPS)	< 4	10 (40.0%)	12 (48.0%)	0.569
	≥ 4	15 (60.0%)	13 (52.0%)	
Vomiting	Yes	15 (60.0%)	15 (60.0%)	1
Dehydration	None	6 (24.0%)	4 (16.0%)	0.758
	Some	12 (48.0%)	14 (56.0%)	
	Severe	7 (28.0%)	7 (28.0%)	
Abdominal distension	None	8 (32.0%)	11 (44.0%)	0.346
	0.5-1 cm	7 (28.0%)	3 (12.0%)	
	> 1 cm	10 (40.0%)	11 (44.0%)
Perianal rash		5 (20.0%)	4 (16.0%)	0.713
Parasite (Giardia)		2 (8.0%)	4 (16.0%)	0.667

At baseline, and day one, diarrhea, vomiting, abdominal pain, abdominal distension, and perineal rash were statistically similar among children of both study groups (Table-II). At day five, lactase enzyme children showed significantly less episodes of diarrhea versus lactose free formula (20.0% vs. 48.0%, p=0.037), while all other complaints were statistically similar and details are shown in Table-II.

**Table-II:** Comparison of complaints between the two groups on baseline, day 1, and day 5 (N=50)

Complaints	Baseline			Day 1			Day 5		
	Lactase enzyme (n=25)	Lactose free formula (n=25)	P- value	Lactase enzyme (n=25)	Lactose free formula (n=25)	P- value	Lactase enzyme (n=25)	Lactose free formula (n=25)	P- value
Diarrhea	25 (100%)	25 (100%)	1	25 (100%)	25 (100%)	*value*	5 (20.0%)	12 (48.0%)	0.037
Vomiting	15 (60.0%)	15 (60.0%)	1	15 (60.0%)	15 (60.0%)	1	2 (8.0%)	6 (24.0%)	0.123
Abdominal pain	15 (60.0%)	13 (52.0%)	0.569	15 (60.0%)	13 (52.0%)	0.569	3 (12.0%)	5 (20.0%)	0.44
Abdominal distension	10 (40.0%)	11 (44.0%)	0.346	10 (40.0%)	11 (44.0%)	0.346	2 (8.0%)	3 (12.0%)	0.637
Perianal rashes	5 (20.0%)	4 (16.0%)	0.713	5 (20.0%)	4 (16.0%)	0.713	1 (4.0%)	2 (8.0%)	0.6

The duration of hospital stay was significantly less among children in lactase enzyme group versus lactose free formula group (p=0.001). Stool for reducing substances (p=0.069), and stool pH (p=0.069) were statistically similar children of both study groups, and the details are shown in Table-III.

**Table-III:** Comparison of final outcomes in two groups on day 6 of treatment (N=50)

Parameters		Frequency (% age)		P-value
		Lactase enzyme (n=25)	Lactose free formula (n=25)	
Stool for reducing substances	-ve	20 (80.0%)	14 (56.0%)	0.069
	+ve	5 (20.0%)	11 (44.0%)	
Stool pH	> 6	20 (80.0%)	14 (56.0%)	0.069
	≤ 6	5 (20.0%)	11 (44.0%)	
Hospital stay (days) mean±SD		4.04±1.05	5.64±1.84	0.001

## DISCUSSION

This study revealed that diarrhea (100%), vomiting (60.0%), abdominal pain (44.0%), and abdominal distension (62.0%) were the most pronounced symptoms in persistent diarrhea, showing similar findings to the study of Yousuf et al.^14^ done at a local hospital on lactose-intolerant stunted children under five years of age secondary to enteric infections. Exploring a total of 50 patients, it was observed that the response of lactase enzyme supplementation was superior to lactose free formula in relation to a faster recovery (hospital stay; 4.04±1.05 vs 5.64±1.84 days, p=0.001). A double-blind case-control study to assess the role of the lactase enzyme in persistent diarrhea among the pediatric population of age range 1-12 months demonstrated that 76% of the patients were symptom-free at the end of the study, whereas in the placebo group only 23% were symptom-free revealing the superiority of the lactase enzyme as compared to the placebo, which aligns with the present research research.^13^ The pooled results of a meta-analysis of 2 RCTs conducted on children under 36 months of age with persistent diarrhea without severe malnutrition showed a significant reduction in the risk of treatment failure in patients receiving lactose-free and semi-elemental formulas compared to lactose-containing feeds (relative risk = 0.17; 95% CI: 0.06 to 0.48; p=0.001). However, authors considered it to be low evidence, probably due to a lack of supportive data regarding the outcomes of diarrhea duration and stool output.^15^ More than half the patients in this study responded to lactose-free formula, which is significantly higher than a retrospective analysis carried out in children under five years of age with persistent diarrhea using a WHO-recommended stepwise diet algorithm.^16^ The median days for clinical response and hospital stay were significantly higher compared to this study, probably due to a comparatively larger sample size.

Medow et al.^17^ conducted a study on the pediatric population to determine the hydrolytic capability of lactase-containing tablets taken immediately prior to an oral lactose challenge. Participants ingested one tablet of β-galactosidase per 5g of lactose immediately before the carbohydrate challenge, and outcomes were compared with the placebo group. It was reported that hydrogen production was considerably larger following placebo (maximum hydrogen excretion, approximately 60 ppm) in contrast to lactase-containing tablets (maximum hydrogen excretion, 7 ppm) and was associated with clinical symptoms of abdominal pain (89% of subjects following placebo ingestion), bloating (83%), diarrhea (61%), and flatulence (44%). These results indicate that co-ingestion of lactose and lactase-containing tablets meaningfully decreases breath hydrogen excretion and clinical symptoms linked to lactose intolerance. Another placebo-controlled study conducted on children with primary lactase deficiency demonstrated a significant reduction in hydrogen breath excretion as well as improvement of symptoms of lactose intolerance in these subjects when oral lactase enzyme was ingested immediately prior to a lactose-containing meal.^18^

Numerous researchers have used oral lactase enzyme in infantile colic with good results, translating to reduced crying time and adequate weight gain.^19^-^21^ The present study was one of its kind, to the best of our knowledge, that entails the comparison between lactose-free formula and oral lactase enzyme. Other studies were conducted comparing lactose-free formula with lactose-containing diets or placebos, but not with oral lactase enzyme in the pediatric population with lactase deficiency secondary to enteric infections. Oral lactase enzyme has been used mainly in adult hypolactasia, with very little work in pediatric primary hypolactasia, but hardly any comparative studies in pediatrics with lactose-free formula. The present findings suggested that oral lactase enzyme in comparison to lactose-free formula was better. Although the lactase enzyme has been in use since the end of the last century for various ailments, its use as yet in the territory of children with lactose intolerance secondary to gastrointestinal infections is optimally unchartered with a paucity of current studies and as such mandates that more research is conducted using oral exogenous lactase enzyme supplementation. Further research in this area could revolutionize management and pave the way for much-needed guidelines for the same, as this would aid faster recovery and prevent children from tipping into the quicksand of malnutrition and ultimately death from current or subsequent infections that are a complication of the former.

### Limitations:

The main limitation of this study was its relatively small sample size. Stool for reducing substances and pH have poor sensitivity and is not recommended to be used for disaccharidase deficiencies.^22^ Although it is not the gold standard, it was used only because it was not feasible for the under two years old children to fast for the required almost 12 hours prior to the test. Currently only a couple of settings in our city are conducting the hydrogen breath test, which is very costly, since being a resource-poor country, the patients belonged to the very low socioeconomic bracket.

## CONCLUSION

This study concludes that lactase enzyme supplementation response was better than lactose-free formula in children aged 6-24 months with persistent and severe persistent diarrhea due to secondary lactose intolerance. The recovery period was faster in children using lactase enzyme versus lactose free formula.
